# Inhibition of bacterial and human zinc-metalloproteases by bisphosphonate- and catechol-containing compounds

**DOI:** 10.1080/14756366.2021.1901088

**Published:** 2021-03-23

**Authors:** Fatema Rahman, Tra-Mi Nguyen, Olayiwola A Adekoya, Cristina Campestre, Paolo Tortorella, Ingebrigt Sylte, Jan-Olof Winberg

**Affiliations:** a Department of Medical Biology, Faculty of Health Sciences, UiT-The Arctic University of Norway, Tromsø, Norway; b Department of Pharmacy, Faculty of Health Sciences, UiT-The Arctic University of Norway, Tromsø, Norway; c Department of Pharmacy, University of “G. d’Annunzio” Chieti, Chieti, Italy; d Department of Pharmacy, Science of Pharmacy, University “A. Moro” Bari, Bari, Italy

**Keywords:** Zinc proteases, bacterial virulence factors, matrix metalloproteases, enzyme inhibition, docking and scoring

## Abstract

Compounds containg catechol or bisphosphonate were tested as inhibitors of the zinc metalloproteases, thermolysin (TLN), pseudolysin (PLN) and aureolysin (ALN) which are bacterial virulence factors, and the human matrix metalloproteases MMP-9 and −14. Inhibition of virulence is a putative strategy in the development of antibacterial drugs, but the inhibitors should not interfere with human enzymes. Docking indicated that the inhibitors bound MMP-9 and MMP-14 with the phenyl, biphenyl, chlorophenyl, nitrophenyl or methoxyphenyl ringsystem in the S_1_′-subpocket, while these ringsystems entered the S_2_′- or S_1_ -subpockets or a region involving amino acids in the S_1_′- and S_2_′-subpockets of the bacterial enzymes. An arginine conserved among the bacterial enzymes seemed to hinder entrance deeply into the S_1_′-subpocket. Only the bisphosphonate containing compound RC2 bound stronger to PLN and TLN than to MMP-9 and MMP-14. Docking indicated that the reason was that the conserved arginine (R203 in TLN and R198 in PLN) interacts with phosphate groups of RC2.

## Graphical Abstract

**Figure:**
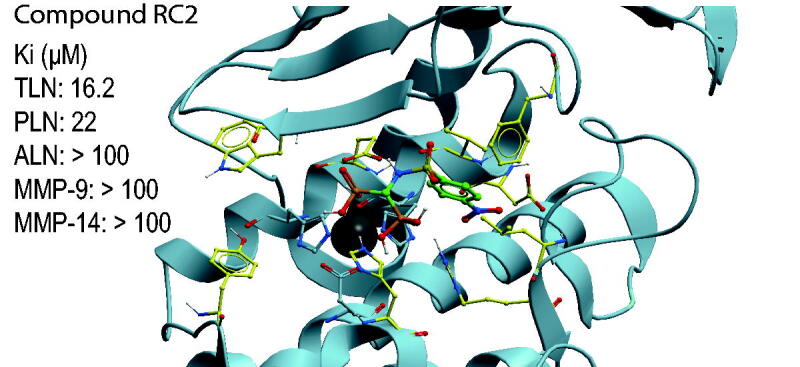

## Introduction

It is estimated that there are more than 60000 different proteases^1^^,^^2^. In vertebrates they are involved in regulation of physiologic processes such as cell growth, angiogenesis, blood pressure, coagulation, cell signalling, reproduction, wound repair, hemostasis and homeostasis^2–7^. Proteases are either secreted from cells or localised inside cells. They are divided into classes and clans depending on the active site residues taking part in the catalytic reaction (Merops database)^8–10^. The major classes found in all organisms are aspartate-, threonine-, cysteine-, serine- and metallo-proteases, but in addition the classes glutamate-, aspargine- and mixed-proteases have been detected in microorganisms (Merops database)^10^.

Dysregulation of one or several proteases in humans is often associated with disease^3^^,^^4^^,^^11–14^ and several proteases are potential targets for therapeutic interventions^15–18^. In humans there are around 570 different proteases and approximately 190 of these are metalloproteases^19^. Of around 280 cell-secreted human proteases, approximately 120 are metalloproteases^2^^,^^19^^,^^20^. Matrixins or matrix metalloproteases (MMPs) is a family of secreted and membrane associated calcium dependent metalloproteases which contains a catalytic and a structural zinc ion^11^. MMPs belong to the M10 family of proteases. In humans there are 23 different MMPs, and MMP-9 and -14 are two of the members^11^. One or several members of the MMP family are overexpressed and functionally involved in pathological conditions such as chronic venous disease, fibrotic disorders, inflammation, liver diseases, lung diseases, neurological diseases, osteoarthritis, viral infection, cardiovascular diseases and in various cancer forms^21^. Several investigators both in academia and industry have developed MMP inhibitors interacting with the active site. However, in clinical tests the vast majority of MMP inhibitors have failed^22^ The most likely reason is that the MMPs are of major importance in many physiological processes such as cell apoptosis, embryogenesis, immune response, morphogenesis, tissue remodelling, tooth enamel formation, reproduction, menstruation, wound healing, angiogenesis and axonal growth^13^^,^^21^^,^^23^. MMPs are tightly regulated and expressed in all human tissues and organs^13^^,^^22^^,^^23^, and therefore an uncontrolled activity regulation of one or several MMPs by an inhibitor should be avoided. In microorganisms, proteases are involved in processes such as generation of nutrition, growth, survival and invasion into host organisms^24–30^. Bacterial infectious diseases claim millions of casualties each year, and the spreading of antibiotic multi-resistance among central human pathogenic bacteria is recognised as a major global health concern and a pressing societal challenge. Development of new antibiotics with novel modes of action and innovative strategies to efficiently fight bacterial infections are urgently needed. Inhibition of bacterial virulence rather than directly targeting bacterial growth and viability has gained increasing interests in anti-infective drug discovery^31^^,^^32^. Such compounds may impose less evolutional pressure for resistance development than classical antibiotics, and have limited impact on the host commensal flora. Several proteases are bacterial virulent factors and therapeutically interesting as putative antibacterial drug targets^26–29^^,^^33^. However, compounds targeting bacterial virulence have so far not been approved as drugs^34^^,^^35^.

MMP-9 (gelatinase B) is secreted, while MMP-14 belongs to the membrane type metalloproteases (MT-MMPs), and is also called membrane type 1 metalloprotease (MT1-MMP). The MT-MMPs contain either a transmembrane domain or a GPI-membrane anchor, with the catalytic site located outside the cell in the extracellular environment^11^. The MMPs are constituted of different structural domains, and both MMP-9 and MMP-14 contain an N-terminal prodomain, followed by a catalytic domain, a hinge region and a C-terminal hemopexin like (HPX) domain. In MMP-14, the HPX domain is followed by the transmembrane domain, while MMP-9 contains three fibronectin-II like (FnII) repeats in the catalytic domain^11^. The MMPs belong to the clan metzincins and the catalytic zinc ion is bound to the protein through three histidines of the segment (HEXXHXXGXXH/D + M)^8^^,^^9^. The fourth zinc ligand in the inactive proform is the cysteine in the PRCGV motif of the pro-domain^36^^,^^37^. The fourth zinc ligand in the activated MMPs is a water molecule, that also binds to the side chain of the glutamate that follows the first histidine in the zinc binding segment^8^^,^^9^. MMP-9 can be activated in the extracellular environment by naturally occurring proteases such as trypsin, kallikrein, MMP-2 and MMP-3, but also by mercurial and organomercurial compounds such as HgCl_2_ and APMA (p-aminophenylmercuric acetate) and bacterial metalloproteases such as thermolysin (TLN) and pseudolysin (PLN)^37^. MMP-14, like the other MT-MMPs, are activated inside cells by the serine protease furin^11^. MMP-14 is the most studied enzyme among membrane-linked MMPs, while MMP-9 (gelatinase B) is the most studied among secreted MMPs^14^^,^^38^. Binding of inhibitors to the active site of MMP-14 and MMP-9 have been extensively studied both by kinetic and X-ray crystallography^39–47^. The active sites of the MMPs are similar but not identical. Their S_1_'-subpocket determines the substrate cleavage site, and they all prefer hydrophobic amino acids in this pocket^48^^,^^49^.

TLN from *Bacillus thermoproteolyticus* is the model enzyme of the M4 family of proteases, which is also termed the thermolysin family^50^. These enzymes have a zinc ion in the catalytic site, which has tetrahedral coordination. Two histidines of a HEXXH motif and a glutamic acid located 18–72 residues C-terminal to the HEXXH motif are the three ligands that anchor the zinc ion to the enzyme, while the fourth ligand is a water molecule as in the MMPs, which also binds the side chain of the glutamate following the first histidine in the zinc binding segment^8^^,^^9^^,^^50^. Inhibitors containing a metal binding group replace the catalytic water molecule on the zinc ion when they bind the catalytic site^51^. TLN, PLN from *Pseudomonas aeruginosa* (LasB or elastase of *P. aeruginosa*) and aureolysin (ALN) from *Staphylococcus aureus* belong to the subclan MA(E) of the M4 family, also known as the “Glu-zincins”^8^^,^^9^^,^^50^. These three proteases have several similarities despite a modest sequence identity (28% between TLN and PLN)^52^^,^^53^. The three dimensional (3D) structures of PLN and TLN have been extensively studied, also in complex with inhibitors, and reveal large similarities in the overall structure. The main structural differences are that PLN consists of a slightly more open substrate binding cleft than TLN, and that PLN has one structural calcium while TLN has three^53–55^. For ALN only the 3 D-structure of the free enzyme is known^56^. Although PLN is not as well characterised as TLN, it appears that the slight difference in substrate specificity between the two enzymes is mainly due to the size of the S_1_′-subpocket and a more open substrate binding cleft in PLN than in TLN. PLN has a broader substrate specificity than most other M4 family members including TLN, although all these enzymes prefer a hydrophobic amino acid at the P_1_’ position. Furthermore, for substrate degradation four subsites of PLN require to be occupied^50^^,^^53^^,^^55^.

PLN, TLN and ALN are secreted bacterial virulence factors, and inhibitors may be new antibacterial drugs, either alone or used as adjuvant to traditional antibacterial treatment. PLN, TLN and ALN have structural resemblance with human MMPs. In order to have a therapeutic value, compounds targeting these virulence factors should not interfere strongly with the function of human MMPs, due to the importance of MMPs in physiological processes. Identifying structural determinants for strong binding to the bacterial M4 proteases and human MMPs would therefore be of pivotal importance for the development of new antibacterial drugs. In this study, we have studied several catechol containing and bisphosphonate containing compounds for their inhibition of TLN, PLN, ALN, MMP-9 and MMP-14 in order to identify new M4 inhibitors and investigate structural determinants that might be important for selective binding using inhibition kinetic and molecular modelling.

## Results and discussion

### K_m_ values for the fluorescence quenched substrates

At conditions used in the present work (1% DMSO in all assays), the *K*_m_ values of the substrate McaPLGL(Dpa)AR-NH_2_ with APMA-activated recombinant MMP-9 (rMMP-9(A)), trypsin-activated MMP-9 from THP-1 cells (MMP-9(T)) and MMP-14 were 4 ± 1, 6 ± 2 and 4.9 ± 0.4 µM, while the *K*_m_ values of the substrate McaRPPGFSAFK(Dnp)-OH with ALN, PLN and TLN were 76 ± 7, 24 ± 8 and 6 ± 1 µM, respectively. The estimated K_m_ values for ALN and PLN must be regarded as uncertain since the highest substrate concentration used was 10 µM due to quenching. The obtained *K*_m_ values are very similar to those previously obtained for theses enzymes without DMSO^57^ or with a DMSO concentration of 5%^58^. Previously it was reported that TLN is inactivated in most organic cosolvents, but can tolerate up to 10% DMSO to enhance substrate solubility^59^. For TLN, the *K*_m_ value was also determined with a DMSO concentration of 2%. This resulted in a *K*_m_ value of 7 ± 1 μM which is not significantly different from the value obtained in the presence of 1% DMSO.

### Quenching experiments with catechol containing compounds and bisphosphonates

All compounds were first tested for possible quenching of the formed fluorescence product. The experiments were performed with varying concentrations of the putative inhibitors (0 − 100 μM) against varying fluorescence product (McaPL-OH) concentration as previously described for PAC-1 and Isatin derivatives^58^, and as described in the experimental section. These experiments revealed that in contrast to the PAC-1 and Isatin derivatives^58^, neither the catechol derivatives nor the bisphosphonates used in the present work quenched the fluorescence product. However, some of the compounds showed fluorescence at the emission and excitation wavelength used, but that did not affect the inhibitory assays as enzymatic reactions were followed continuously.

### Inhibitory effects of catechol containing compounds

**Figure 1.**
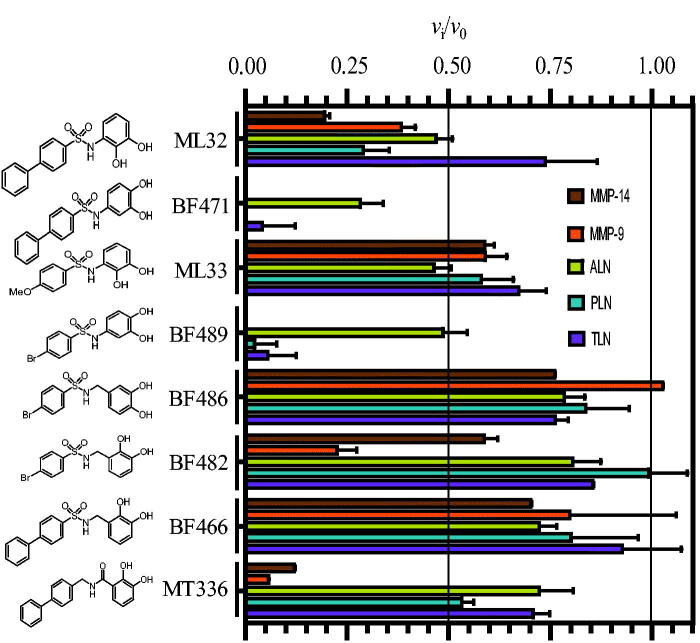
Inhibitory effect of 100 μM of the catechol containing compounds on the activity of the human metalloproteases, MMP-9 and MMP-14, and the bacterial metalloproteases TLN, PLN and ALN. The inhibition experiments were performed by using a fixed concentration of 4 μM of both the MMP-9 and MMP-14 substrate McaPLGL(Dpa)AR-NH_2_ and the ALN, PLN and TLN substrate McaRPPGFSAFK(Dnp)-OH. The *v*_i_/*v*_0_ (mean ± sd) were based on 4–6 experiments.

The inhibitorial power of 100 μM of the eight catechol containing compounds was tested against the two human metalloproteases MMP-9(T) and MMP-14 and the three bacterial metalloproteases ALN, PLN and TLN (Figure 1). In order to detect putative slow and slow-tight binding, the catechol derivatives were first incubated along with the enzyme for 15 min at 37 °C. In controls without inhibitor present, enzymes and buffer were preincubated under identical conditions. Thereafter, the enzyme reaction was started by adding the relevant chromogenic substrate and the rate was followed continuously for 30 min. Except for BF486, which did not affect MMP-9, and BF482 which did not affect PLN, all cathecol containg compounds showed inhibition of the five proteases (Figure 1). Both BF471 and BF489 showed more than 50% inhibition of all five proteases, while ML32 reduced the activity more than 50% for four of the proteases (not for TLN). The activity was reduced with more than 50%, by MT336 for the two MMPs, by BF482 for MMP-9(T) and by ML33 for ALN (Figure 1). In all other inhibitory studies with catechol containing compounds, the activity was reduced between 0 and 45%.

X-ray crystallography studies showed that BF471 binds to the active site of MMP-8^60^, and hence we could expect that all tested catechol derivatives should bind the active site. To assure that this is correct, the inhibitors BF471 and BF489 were tested against varying McaRPPGFSAFK(Dnp)-OH using TLN as described in the Experimental section. The results showed that the two catechol derivatives competed with the substrate (data not shown) and the *K_i_* values obtained were 57 ± 6 μM for BF471 and 73 ± 11 μM for BF489. It should be noted that the reaction was started by adding the enzyme to the substrate-inhibitor mixtures. In the case of slow binding, the full potential of these inhibitors would not be realised by this inhibitory assay. When the activity was reduced by more than 60%, experiments were performed with varying concentrations of the catechol derivatives. IC_50_ values were obtained from dose response plots, and *K_i_* values were determined from the IC_50_ values based on substrate competitive inhibition. By the use of *K_i_* values, we can compare the binding strength of the compounds for the different enzymes and not only comparing the compounds ability to bind one enzyme. The obtained *K_i_* values of the catechol contaning compounds for the five tested proteases are given in Table 1. There are several possible explanations for the deviation in determined *K_i_* value of BF471 and BF489 for TLN from the dose response plots and the double inverse plots. One possible explanation is that the catechol compounds are slow binders, and hence less inhibitory activity is observed without preincubation of enzyme and inhibitor.

**Table 1.** *K_i_* values of the catechol containing compounds for TLN, PLN, ALN, MMP-14, and MMP-9(T).

Compound	*K_i_* ± sd (μM)				
	McaRPPGFSAFK(Dnp)-OH			McaPLGL(Dpa)AR-NH_2_	
	TLN	PLN	ALN	MMP-14	MMP-9(T)
ML32	N.D.	38 ± 8	N.D.	19 ± 0.8	51 ± 17
BF471	13 ± 2	9 ± 3	49 ± 5	6.6 ± 0. 6	13 ± 2
BF489	14 ± 5	16 ± 3	N.D.	8.3 ± 0.6	12.6 ± 0.6
MT336	N.D.	N.D.	N.D.	12 ± 1	13 ± 1

*K_i_* values determined for both bacterial and human metalloproteases using two different fluorescence quenched peptide substrates, McaRPPGFSAFK(Dnp)-OH (for TLN, PLN, and ALN) and McaPLGL(Dpa)AR-NH_2_ (for MMP-9(T) and MMP-14). The concentration of the substrates used was 4 µM and the highest concentration of tested inhibitors was 100 µM. Compounds included are only those where 100 µM of the compound reduced the enzymatic activity by 60% or more as described in the text. The *K_i_* values are based on 4–6 experiments.

N.D.: not determined.

Figure 1 and Table 1 show that the position of the OH-groups in the catechol moiety affects the binding. Moving the OH-group from position 2 to position 4 (ML32 vs. BF471 and ML33 vs. BF489) largely strengthen the binding for four of the enzymes, while the effect was less for ALN. Another striking effect occurs with the addition of a methylene group between the catechol moiety and the sulphonamide group, which resulted in weaker binding (BF489 vs. BF486 and ML32 vs. BF466). Changing the sulphonyl group to a methylene group and the methylene between the catechol moiety and the sulphonylamide moiety to a carbonyl (BF466 vs. MT336) resulted in stronger binding for the two MMPs, but with only limited activity changes for the bacterial enzymes. Overall, it appeared that the structural differences between the catechol derivatives had little effect on the ALN activity, while the activity of the other four enzymes varied correspondingly. None of the catechol derivatives showed stronger binding to the bacterial than the human enzymes. The largest differences in binding strengths between the human MMPs and the bacterial proteases were seen for MT336.

**Figure 2.**
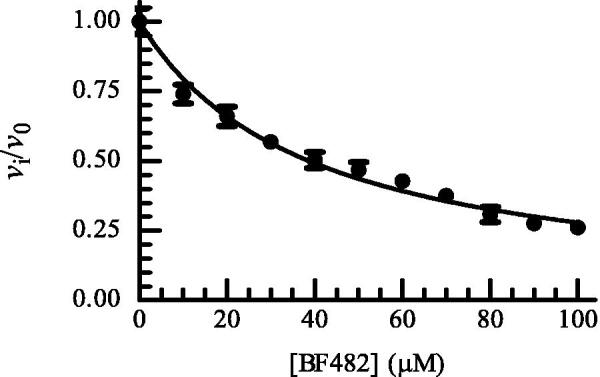
Dose response plot of BF482 for MMP-9(A). The enzyme with and without inhibitor was pre-incubated for 30 min at room temperature and the reaction was started by adding McaPLGL(Dpa)AR-NH_2_ (4 μM in assay). The reaction was allowed to proceed for 4 h at 37 °C and stopped by the addition of EDTA (10 mM end concentration). The relative fluorescence was determined with the Clariostar plate reader as described in the Experimental Section, where *v*_i_ and *v*_0_ are the reaction rates in the presence and absence of BF482, respectively. Each point on the curve shows the mean ± sd (*N* = 5 for all points except for two points where *N* = 4). The regression coefficient *r*^2^ is 0.97, with a determined IC_50_ value of 38.8 ± 0.9 μM and a *K_i_* value of 19.4 ± 0.4 μM.

Except for ML33, all catechol derivatives have previously been tested for binding to MMP-2, MMP-8 and MMP-9^60^. For six of them, IC_50_ values for the enzymes between 2 and 12 µM were reported. The exception was MT336, which had IC_50_ values between 4 and 56 µM. The *K_i_* values for MMP-9 in the present study were higher than the IC_50_ values in the previous study, the exception was MT336 for which we obtained a lower value. It is not easy to point out a single factor in the experiments contributing to deviations between the studies. Both studies used a pH of 7.5 but the buffer compositions were slightly different. In the present study, we used MMP-9 purified from THP-1 cells and activated by trypsin, and hence the enzyme has its C-terminal hemopexin domain intact^57^. Tauro et al.^60^ used a commercial active MMP-9 produced in *E-coli* that only contained the catalytic domain and the fibronectin-like (FnII) module. We do not believe that the use of the two different variants of MMP-9 should affect binding of the catechol derivatives, as we previously have shown that small MMP inhibitors, like galardin and an azasugar-based hydroxamate compound had, the same strength of binding to different N- and C-terminal truncated variants of recombinant MMP-9 and trypsin activated MMP-9 from THP-1 cells^57^. A factor that may contribute to differences is that after preincubation of MMP-9 with catechol derivatives and adding of the substrate for starting the reaction, we followed the reaction continuously for 30 min, while Tauro et al.^60^ used an endpoint assay allowing the reaction to proceed for 2 to 4 h before the fluorescence was measured. We did some tests to determine if some of these differences could affect the binding results. In the test experiments, we used rMMP-9(A) which differs from the MMP-9(T) by a slightly different N-terminal and a largely truncated C-terminal HPX domain^57^. One hundred µM of BF466 was tested where the 0.1 M Hepes buffer pH 7.5 was exchanged to a 0.1 M Tris-HCl buffer pH 7.5. The rMMP-9(A) was preincubated with BF466 for 0, 15 and 30 min at 37 °C and the reaction was started by the addition of the substrate McaPLGL(Dpa)AR-NH_2_ (4 µM in the assay) following the reaction continuously for 3 h. The controls without BF466 were treated identically. The obtained *v*_i_/*v*_0_ values for the three time points were 0.74 ± 0.04 (*N* = 4), 0.68 ± 0.02 (*N* = 4) and 0.73 ± 0.05 (*N* = 4). These results fit well with obtained data for MMP-9(T) in Hepes buffer (Figure 1) suggesting that neither the buffer nor the origin of the MMP-9 affected the binding of this inhibitor. Further, the compound could not be regarded as a slow binder of MMP-9. We also tested if the use of an end point assay could affect the results. Here rMMP-9(A) was preincubated with different concentrations of BF482 for 30 min in 0.1 M Hepes pH 7.5 and the reaction was started by the addition of McaPLGL(Dpa)AR-NH_2_ (4 µM in the assay). The reaction was allowed to proceed for 4 h, and the reaction was stopped by addition of EDTA (end concentration 10 mM) and the relative fluorescence intensity determined. This resulted in an IC_50_ value of 40 ± 1 µM and a *K_i_* value of 20.1 ± 0.7 µM (Figure 2), while the *v_i_/v_0_* value for 100 µM inhibitor was similar to that in Figure 1. This suggests that the use of either initial rate assays or end point assays is not a reason for the obtained differences between this study and that of Tauro et al.^60^.

#### Docking

**Figure 3.**
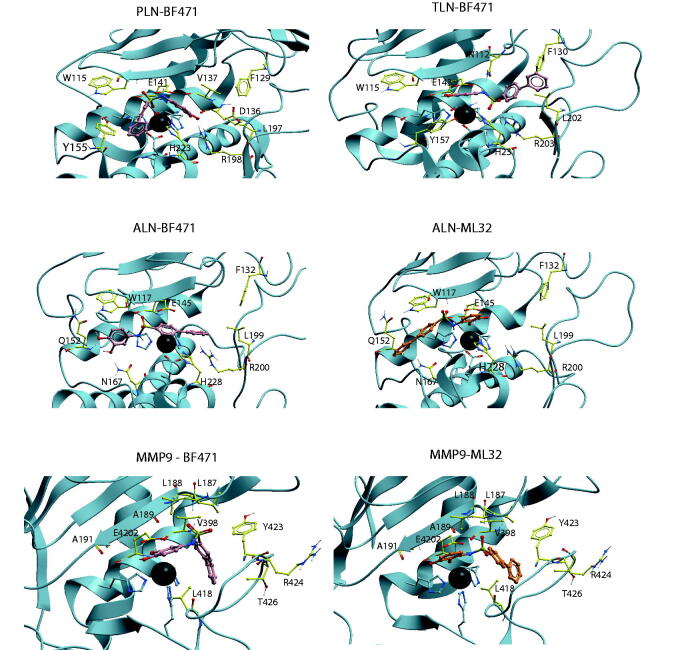
BF471 docked into PLN, TLN, ALN and MMP-9, and ML32 docked into ALN and MMP-9. Zinc is shown in dark grey. The side chain of zinc coordinating amino acids are shown in blue. The side chains of some amino acids of importance for ligand binding are displayed with the following colour coding of atoms: oxygen: red; nitrogen: blue, carbon: yellow, hydrogen: grey. Colour coding of ligand atoms: oxygen: red; sulphur: green; nitrogen: blue; hydrogen; grey; carbon (BF471): pink; carbon (ML32): orange.

Docking of the catechol containing compounds into the X-ray structure of MMP-9 (PDB ID: 5cuh) and MMP-14 (PDB ID: 1bqq) showed that the biphenyl, bromophenyl and methoxyphenyl group of the compounds enter the S_1_' -subpocket. The interaction modes of BF471 in MMP-9 and MMP-14 were very similar to that observed in the X-ray structure of BF471 with MMP-8^60^. In contrast, docking catechol containing compounds into TLN (PDB ID: 5dpe) and PLN (PDB ID: 1u4g) showed docking poses both with the diphenyl, bromophenyl and methoxyphenyl groups entering the S_1_- or the S_1_′/S_2_′ -subpockets. The cathecol containing compounds bind quite weak to ALN, and docking indicated binding modes quite different from the other proteases (Figure 3). It seems like the side chain of R203 in TLN, corresponding to R200 in ALN and R197 in PLN, is the main reason for the binding pose differences between the human and bacterial proteases. For most of the compounds, the arginine side chain orientation hinders the biphenyl, bromophenyl and methoxyphenyl groups of the compounds to enter deeply into the S_1_'-subpocket (Figure 3). This arginine is known as functionally important for TLN-like proteases, and is suggested to interact with a backbone carbonyl group of the substrates^61^ and is located at the border between the S_1_′- and S_2_′-subpockets. Table 2 shows the most important amino acids in the different protease subpockets for binding the compounds in the present study.

**Table 2.** Functionally important amino and amino acids in the S_1-,_ S_1_′- and S_2_′-subpockets of TLN, PLN, ALN, MMP-9, and MMP-14 contributing to the binding of catechol or bisphosphonate containing compounds tested in the present study.

	TLN	PLN	ALN	MMP-9	MMP-14
S_1_-subpocket	W115, Y157	W115, Y155	W117, Q152, N167	H190, P193	F204
S_1_′-subpocket	L133, V139, H142, I188	L132, V137, H140, I190	V137, H144, I186, V189	L397, V398, H401, L418, Y420, P421, Y423, R424, T426	L199, E219, W221, N231, H239
S_2_′-subpocket	N111, F130, Y193, L202	E111, F129, L197	N114, F132, L199	G186, L187, Y218	L235, V236, Y261, Q262
Catalytic Glu	E143	E141	E145	E402	E240
Known inhibitor binding residues	N112, A113, F114, R203, H231	E111, N112, A113, R198, H223	N112, Y114, R200, H228	A189, H190, A191	A200, H201
Zn^2+^ ligated	H142, H146, E166	H140, H144, E164	H144, H148, E168	H401, H405, H411	H239, H243, H248

The subpocket amino acids of MMP-9 in the table are based on the X-ray structure of the inactive MMP-9 mutant E402A lacking the pro, FnII, hinge and HPX domains bound to a chromogenic substrate (PDB id: 4JIJ)^71^, while the numbering is as in the PDB id: 1l6j, which includes the three FnII-like repeats in the catalytic site^72^. The subpocket amino acids of TLN are based on Krimmer et al.^73^. Subpocket amino acids of the other enzymes are based on structural superimposition with TLN (PLN and ALN) and MMP-9 (MMP-14).

The highest scored pose of BF471 with TLN was with the diphenyl moiety into the S_2_′-subpocket, with the sulphonamide group interacting between the side chain of R203, the side chain of N112 in the S_2_'-subpocket and H231 (Figure 3). Both hydroxyl groups of the BF471 catechol ring interacted with the Zn atom, while the hydroxyl group in position 3 also created a hydrogen bond with E143. However, the best pose of BF489 in TLN had the bromophenyl entirely into the S_1_′-subpocket, while a hydroxyl of the catechol ring interacted with the backbone of W115 (S_1_-subpocket) and the amide with E143, thereby obtaining a binding pose quite similar to that observed for BF471 and the other catechol containing compounds in MMP-9 and MMP-14 (Figure 3).

The highest scored pose of BF471 in PLN was with the diphenyl moiety into the S_1_-subpocket interacting close to Y155, while the catechol moiety entered into the region between the S_1_′- and S_2_′-subpocket with the hydroxyl groups interacting with R198 (Figure 3). The NH-group formed a hydrogen bond with E141. Moving the hydroxyl group from position 2 (ML32) to position 4 (BF471) of the catechol moiety resulted in increased binding affinity towards all enzymes. In PLN the change allowed interactions between both hydroxyl groups of BF471 and R198, while only one hydroxyl group of ML32 interacted with R198 which may explain the higher PLN affinity of BF471 than of ML32.

The compounds showed weak affinity for ALN (Figure 1), and only BF471 reduced the enzymatic activity with more than 60% at a concentration of 100 µM and a *K_i_* value of 49 μM was determined. BF471 obtained a docking pose in ALN quite different from those in the other enzymes (Figure 3). The catechol hydroxyl groups formed hydrogen bonds with Q152 and N167 in the S_1_-subpocket, while the NH group formed hydrogen bonds with the backbone of W117 and with the biphenyl ring-system located above the Zn^2+^and R200. The possibility of two hydrogen bonds (with Q152 and N167) is perturbed for ML32 with the hydroxyl group in position 2 (Figure 3). Instead the hydroxyl groups of ML32 were occupied with Zn^2+^, while diphenyl ring system was highly exposed to solvent without clear interactions with amino acids in ALN. Based on the docking it was not easy to explain the increased binding affinity of BF471 for ALN compared with ML32. Both compounds docked quite similar into the enzyme. However, the meta-hydroxyl group (position 3) of ML32 was located further from Y157 in the S_1_-subpocket without the possibility of a hydrogen bond, and in addition the sulphonamide group in ML32 was located further from H231 than the corresponding group in BF471.

Docking indicated that ML32 and ML33 bound similarly to MMP-9 and -14. The hydroxyl group in position 2 of both compounds formed hydrogen bonds with the two oxygen atoms of the side chain carboxyl group of E402 (MMP-9 numbering). The hydroxyl group in position 2 seems to replace the zinc-bound water in the free enzyme, as it also interacted with the catalytic zinc. The two oxygen atoms at the sulphonylamide group formed hydrogen bonds with the main chain NH groups of L187 and A189, while the amide hydrogen of the compounds formed hydrogen bonds with the main chain carbonyl of P421. Thus the main difference in binding strength between ML32 and ML33 for MMP-9 and -14 is mainly attributed to the difference in interaction with the S_1_'-subpocket by the diphenyl and the methoxyphenyl group.

The position of the OH– groups in the catechol in relation to the position of the amide nitrogen bound to the catechol seemed important for inhibitory capacity of the compounds. Moving the hydroxyl in position 2 of ML32 and ML33 into position 4 (BF471 and BF489) seemed to strengthen binding to MMP-9 and MMP-14 (Table 1, Figure 1). BF471 docked into MMP-9 with the hydroxyl group in position 3 forming a hydrogen bond with the side chain of E402, while the hydroxyl group in postion 4 interacted with the backbone CO of A191. Both hydroxyl groups of BF471 and BF489 also interacted with Zn^2+^, while only the hydroxyl group in position 2 of ML32 and ML33 interacted with Zn^2+^. Introduction of a methyl group between sulphonylamide moiety and the catechol ring of BF489 giving compound BF486 resulted in decreased inhibition of all enzymes (Figure 1). Docking indicated that the bromophenyl group of BF486 was deeper into the S_1_'-subpocket of MMP-9 and -14 than that of BF489, and one of the oxygen at the sulphonylamide group formed a hydrogen bond with the main chain nitrogen of Y423 and not with A189 and L188 as seen for BF489. Furthermore, one side chain carbonyl oxygen of E402 formed a hydrogen bond with the NH group of the sulphonylamide moiety of BF486, while the other E402 carbonyl oxygen interacted with the hydroxyl group in positon 3 of the catechol. In addition, the position 3 hydroxyl group interacted with the backbone CO of A191. These overall changes result in weaker binding of BF486 than of BF489.

MT336 differs from BF466 in that the sulphonyl group of BF466 is replaced by a methylene group, and the methyl group between the sulphonylamide moiety and the catechol ring by a carbonyl group. Hence MT336 contains an amide bond, which improved the binding for MMP-9 and MMP-14 compared to BF466, but not for the bacterial enzymes (Figure 1 and Table 1). Docking into MMP-9 and MMP-14 showed that the carbonyl of MT336 was much closer to the catalytic zinc atom than any of the oxygen atoms of the sulphonylamide moiety of BF466, and in addition, the hydroxyl group in position 2 of the catechol was also closer to Zn^2+^.

### Inhibitory effects of bisphosphonates

**Figure 4.**
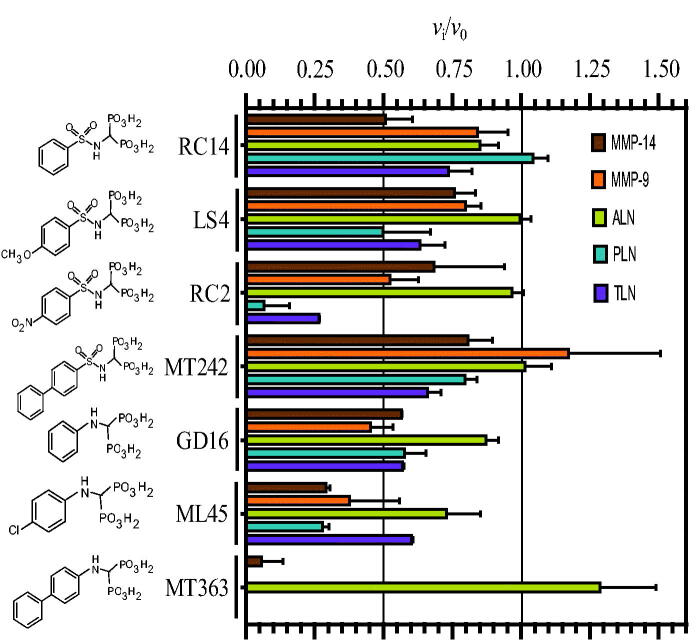
Inhibitory effect of 100 μM bisphosphonate containing compounds on the activity of the human metalloproteases, MMP-9 and MMP-14, and the bacterial metalloproteases TLN, PLN, and ALN. The inhibition experiments were performed by using a fixed concentration of 4 μM of both the MMP-9 and MMP-14 substrate McaPLGL(Dpa)AR-NH_2_ and the ALN, PLN and TLN substrate McaRPPGFSAFK(Dnp)-OH. The *v*_i_/*v*_0_ (mean ± sd) were based on 4–6 experiments.

Figure 4 shows the inhibition of 100 μM of the seven bisphosphonates. Replacing the catechol moiety of ML32 and BF471 with a bisphosphonate giving MT242, and the catechol moiety of ML33 giving LS4, had either no effect or reduced the binding (Figures 1 and 4). Adding a methoxy group to position 4 of the phenyl group (RC14 to LS4) had almost no effect of the binding, except for PLN where the binding was strengthened. The addition of a strong electron withdrawing group (NO_2_) at the position 4 of the phenyl group, giving RC2 resulted in a much stronger binding to PLN and TLN than to the two MMPs (Figure 4 and Table 3). However, for ALN the binding strength was almost similar to that without the NO_2_ group (RC14) or with a methoxy group at the phenyl ring (LS4) (Figure 4). The binding of RC2 to ALN was even weaker than for the two human MMPs (Figure 4). Removal of the sulphonyl group from the bisphosphonate of RC14 giving GD16 had limited effect on the binding of the five proteases (Figure 4). Addition of a chloride ion at position 4 of the phenyl group of GD16 giving ML45 resulted in enhanced binding to all proteases except for TLN (Figure 4 and Table 3). Addition of an additional phenyl ring to position 4 of the phenyl group of GD16, giving MT363, gave much stronger binding for four of the proteases. However, the binding was reduced for ALN (Figure 4 and Table 3). Most bisphosphonates bound weaker to ALN than to the other proteases.

**Table 3.** The obtained *K_i_* values of the various bisphosphonate containing compounds for TLN, PLN, ALN, MMP-14 and MMP-9.

Compounds	*K_i_* ± sd (µM)				
	McaRPPGFSAFK(Dnp)			McaPLGL(Dpa)AR-NH_2_	
	TLN	PLN	ALN	MMP-14	MMP-9(T)
LS4	N.D.	58 ± 4	N.D.	N.D.	N.D.
RC2	16.2 ± 0.4	22 ± 3	N.D.	N.D.	N.D.
ML45	N.D.	37 ± 6	N.D.	17 ± 1	N.D.
MT363	7 ± 1	12 ± 4	N.D.	7.2 ± 0.6	6.6 ± 0.4

The *K_i_* values for both bacterial and human metalloproteases were measured with two different fluorescence quenched substrates, McaPLGL(Dpa)AR-NH_2_ (for MMP-9 and MMP-14) and McaRPPGFSAFK(Dnp)-OH (for TLN, PLN and ALN). The concentration of the substrates used was 4 µM and the highest concentration of the inhibitor compounds tested was 100 µM. Compounds tested were only those where 100 µM of the compound reduced the enzymatic activity by 60% or more. The *K_i_* values are based on 4–6 experiments.

N.D.: not determined.

The seven bisphosphonate compounds were also previously tested against various MMPs including MMP-9 and MMP-14^62^^,^^63^. As for the catechol derivatives, the results for some of the compounds varied slightly from the present results for MMP-9 and MMP-14. A difference between the assays was that in the present study we used a preincubation time of 15 min at 37 °C, while Rubino et al. 2011^62^ and Tauro et al. 2013^63^ used 30 min at 25 °C. Another important difference was that in the present work we followed the reaction continuously for 30 min after preincubation, while Rubino et al. 2011^62^ and Tauro et al. 2013^63^ used an end point assay where the reaction was stopped by adding 3% of acetic acid after 2–4 h of incubation, followed by fluorescence measurements. These differences in assay conditions are not likely explanations for the small differences in the obtained binding values as previously discussed for the catechol derivatives.

#### Docking

**Figure 5.**
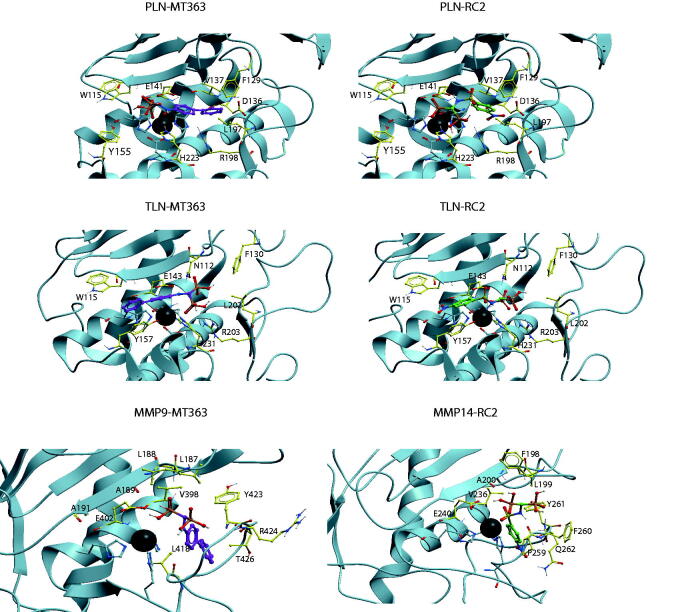
MT363 and RC2 docked into PLN, TLN MMP-9 (MT363) and MMP-14 (RC2). TLN, PLN and MMP-9. Zinc is shown in dark grey. The side chain of zinc coordinating amino acids are shown in blue. The side chains of some amino acids of importance for ligand binding are displayed with the following colour coding of atoms: oxygen: red; nitrogen: blue; carbon: yellow; hydrogen: grey. Colour coding of ligand atoms: oxygen: red; sulphur: green; nitrogen: blue; phosphate: orange; hydrogen: grey; carbon – MT363: purple; carbon – ML32: orange.

Docking indicated that like the catechol containg compounds, the bisphosphonates bind MMP-9 and MMP-14 with the phenyl, biphenyl, chlorophenyl, nitrophenyl or methoxyphenyl ringsystem into the S_1_′-subpocket (Figure 5). The phosphate and sulphonamide groups were located in the region of Zn^2+^, E402, A189, L187, L188, and V398 (MMP-9 numbering). However, docking into TLN and PLN was not conclusive, and docking poses with these ringsystems into the S_1_- or S_1_′/S_2_′-subpockets of TLN and PLN were observed for most of the compounds. Notable features from the binding studies were that RC2 inhibits TLN and PLN stronger than ALN and the two human MMPs, while MT363 is a decent inhibitor of all enzymes, except ALN (Figure 4, Table 3).

Docking of RC2 into PLN showed that one of the phosphate groups interacted with Zn^2+^, H223 and E141, while the other phosphate group interacted with R198 (two hydrogen bonds) and H223. The NH-group interacted with the backbone of A113. The NO_2_-group was located within the S_1_′-subpocket close to the side chain R198, the backbone NH-group of G187 and the side chain of the zinc-coordinating H140 (Figure 5). However, docking poses in PLN with the nitrophenyl group of RC2 in the S_1_-subpocket were also observed. In TLN, poses quite similar to that described in PLN were observed, but the highest scored was with the nitrophenyl group into the S_1_-subpocket interacting with Y157 and with the nitro group exposed to solvent. The sulphonamide group interacted with N112 and H231. One of the phosphate groups interacted with Zn^2+^, and the side chains of R203 and E143, while the other interacted with the side chain of R203 and N112. In MMP-9 and -14, RC2 bound with the the nitrophenyl group into the S_1_′-subpocket (Figure 5). In MMP-14, the phosphate groups were involved in a network of interactions with Zn^2+^ and E240, backbone NH and CO of A200, and the backbone NH groups of L199 and Y261, in addition to being exposed to solvent. The phosphate groups of RC2 had fewer strong interactions with the enzyme in the MMPs than in PLN and TLN, which may contribute to stronger interactions of RC2 with TLN and PLN than with the MMP-9 and -14.

Except for ALN, MT363 binds quite strong to all enzymes. In PLN the biphenyl group of MT363 was located within the S_2_'-subpocket, while one the phosphate groups interacted with Zn^2+^, E141 and the backbone NH of W115, while the other interacted with N112, F114, in addition to being quite solvent exposed. However, docking poses with the biphenyl group into the S_1_-subpocket were also obtained. In TLN, MT363 docked best with the biphenyl group into the S_1_-subpocket obtaining stacking interactions with Y157, while also poses with the biphenyl into the S_2_′-subpocket were obtained. In the best docking pose the phosphate groups interacted with Zn^2+^, R203 and H231, while the other pointed into the S_2_′-subpocket and interacted with N112 (Figure 5). In the MMPs, the biphenyl moiety was in the S_1_′-subpocket, while the bisphosphonate group interacted with Zn^2+^, the side chain of E402 (MMP-9 numbering), V398, the backbone of A189, in addition to being exposed to the solvent.

### Conclusion

Several of the tested MMP-inhibitors were identified as strong TLN and PLN inhibitors, while only BF471 inhbited ALN activity with more than 60%. Both tested catechol containing compounds and bisphosphonates bound MMP-9 and −14 with the the phenyl, biphenyl, chlorophenyl, bromophenyl, nitrophenyl or methoxyphenyl ringsystem into the structurally flexible S_1_′-subpocket. In TLN, PLN and ALN, the contribution of a postively charged arginine (TLN; R203, PLN; R198, ALN; R200) at the entrance of the S_1_′-subpocket hinders that these functional groups fully enter the S_1_′-subpocket of the bacterial enzymes. Instead these groups occupy the S_1_- or the S_2_′-subpocket, or are located at the entrance of the S_1_′-subpocket. However, interactions with the arginine seem to be an important factor for strong binding to the bacterial proteases. RC2- bound stronger to TLN and PLN than to the MMPs. RC2 may be used as a scaffold to identify new compounds that bind much stronger to the bacterial virulence factors TLN and PLN than to the human MMPs, and hence have a therapeutic potential as virulence factor inhibitors and represent a new strategy in the fight against drug resistant bacterial infections.

## Experimental section

### Materials

TRIS, DMSO, Na_2_HPO_4_ and sodium acetate were from Merck (Darmstadt, Germany). EDTA was from Fluka (Buchs, Switzerland). Acrylamide, Commassie Brilliant Blue G-250 and Triton X-100 were from BDH (Poole, UK). RPMI 1640, streptomycin, penicillin, phorbol 12-myristate 13-acetate (PMA), Hepes, Brij-35, Silver nitrate, alkaline phosphatase-conjugated antibodies and gelatine were purchased from Sigma (St Louis, MO, USA). Gelatine-Sepharose, Q-Sepharose and Sephadex G-50 (fine) were from GE-Healthcare (Uppsala, Sweden). DC Protein Assay and unlabelled molecular weight standards were from BioRad (Richmond, CA, USA). Magic Marker molecular weight standards were from Invitrogen (Carlsbad, CA, USA). Western Blotting Luminol reagent and HRP-conjugated donkey anti-goat secondary antibody were from Sancta Cruz (Santa Cruz, CA, USA). HRP-conjugated goat anti-rabbit secondary antibody was from Southern Biotech (Birmingham, AL, USA). Foetal bovine serum was from Biochrom AG (Berlin, Germany). Human MT1-MMP/MMP-14 (catalytic domain), TLN and PLN were from Calbiochem (San Diego, CA, USA) and Aureolysin was from BioCentrum Ltd (Kraków, Poland). McaPLGL(Dpa)AR-NH_2_ (ES001) and McaRPPGFSAFK(Dnp)-OH (ES005) were from R&D Systems (Minneapolis, MN, USA).

### Synthesis of compounds

Synthesis of compounds tested in the present study was reported previously^60^^,^^62^^,^^63^.

### Biosynthesis of proMMP-9

The human leukemic monocyte cell-line THP-1 was a kind gift from Dr. K. Nilsson, Department of Pathology, University of Uppsala, Sweden. The cells were cultured in RPMI 1640 medium with 10% foetal bovine serum, 50 μg/ml of streptomycin, and 100 units/ml of penicillin. To isolate secreted cell-synthesized proMMP-9, the cells were washed 3 times in serum-free medium and then cultured for 72 h in serum-free RPMI 1640 medium with 0.1 μM PMA as described previously^64^^,^^65^. Conditioned medium was harvested, loose cells were pelleted by centrifugation at 1200 rpm (200 g) for 10 min. ProMMP-9 was thereafter isolated and detected as described below.

### Purification and activation of proMMP-9 from the THP-1 cells

The proMMP-9 in conditioned medium from the THP-1 cells was partly purified as described previously^57^^,^^65^^,^^66^. SDS-electrophoresis under reducing conditions, followed by either silver or Coomassie Blue staining, showed two bands, a major band at 92 kDa and a minor band at 28 kDa. Western blotting revealed that the 92 kDa band was proMMP-9, and the 28 kDa band was TIMP-1. The amount of proMMP-9 was estimated spectrophotometrically at 280 nm using ε_280nm_=114,360 M^−1 ^cm^−1^ ^67^, ignoring the contribution of TIMP-1.

The purified proMMP-9 was activated by trypsin, by mixing approximately 300 µg of proMMP-9 with trypsin (31 μg/ml) for 10 min at 37 °C in 0.1 M Hepes pH 7.5, 0.005% Brij35% and 10 mM CaCl_2_. The activation and processing of MMP-9 were terminated by adding a 50 times excess of SBTI (2.7 mg/ml) in relation to trypsin and after 10 min incubation at room temperature, the mixture was transferred and kept on ice during the kinetic and inhibition kinetic measurements. After activation, the activity was determined with 10 µM of McaPLGL(Dpa)AR-NH_2_ in 0.1 M Hepes pH 7.5, 0.005% Brij35% and 10 mM CaCl_2_ in a total assay volume of 100 µL, at 37 °C. Initial rates were measured at an excitation wavelength of 320 nm and an emission wavelength of 405 nm with a slit width of 10 nm using a Perkin Elmer LS 50 Luminescence spectrometer and the FL WinLab Software Package (Perkin Elmer). The amount of active MMP-9 was determined by active site titration using galardin as described previously^57^.

### Expression, purification and activation of recombinant human proMMP-9 in Sf9 insect cells

The expression and purification of recombinant human full-length proMMP-9 (rpMMP-9) from Sf9 insect cells were performed as described previously^57^. The amount of proMMP-9 was estimated spectrophotometrically at 280 nm using ε_280nm_=114,360 M^−1 ^cm^−1^ ^57^. Activation of the recombinant proMMP-9 was performed with APMA (auto-activation) as described previously^57^. The amount of active MMP-9 was determined by active site titration using galardin also described previously^57^.

### Determination of K_m_ values

*K*_m_ values were determined for McaPLGL(Dpa)AR-NH_2_ with APMA-activated recombinant MMP-9 (rMMP-9(A)), trypsin-activated MMP-9 from THP-1 cells (MMP-9(T)) and MMP-14, and for McaRPPGFSAFK(Dnp)-OH with ALN, PLN and TLN. Substrate concentrations used were 1–10 µM in a total volume of 100 µL of 0.1 M Hepes pH 7.5 containing 10 mM CaCl_2_, 0.005% Brij-35 and 1.0% DMSO. Substrate concentrations above 10 µM resulted in quenching as reported previously^58^. Initial rate experiments were performed as described above for the determination of enzyme activity of MMP-9 during activation and the same excitation and emission wavelengths were used for both substrates.

### Determination of IC_50_ and K_i_ values

The various inhibitors were dissolved in 100% DMSO giving an inhibitor concentration of 10 mM. All the inhibitory and control experiments contained a total and fixed concentration of 1.0% DMSO. The inhibitory constant IC_50_ of the various compounds were performed with inhibitor concentrations ranging from 10^−10^ to 10^−4 ^M in the assay, with a fixed substrate concentration of 4.0 µM in a total volume of 100 µL 0.1 M Hepes pH 7.5, 10 mM CaCl_2_, 0.005% Brij-35 and 1.0% DMSO, except for ALN where the substrate concentration was 5.0 µM. The fixed enzyme concentration were as follows; 0.28 nM MMP-9(T), 0.05 nM MMP-9(A), 1.0 nM MMP-14, 1.4 nM ALN, 0.5 nM PLN and 0.21 nM TLN. Enzymes with and without inhibitors were pre-incubated for 15 min at 37 °C, the initial rate assays were started by adding the substrate and the reaction was followed for 30 min. Assays were performed using a Spectra Max Gemini EM micro-plate reader (Molecular Devices) or a Clario Star micro plate reader (CLARIOstar® BMG LABTECH). Assays were performed at 37 °C, using an excitation wavelength of 320 nm and an emission wavelength of 405 nm with a slit width of 10 nm. The IC_50_ values were calculated either in Sigma Plot (Enzyme kinetics 1.3 module) or in Graph Pad Prism 5 using Equations (1) or (2) depending on the concentration span of the used inhibitor:

(1) $$\frac{v_{i}}{v_{0}}=\;\frac{1}{\left(1+\;10^{\left(\mathrm{pIC}50-pI\right)}\right)}$$

(2) $$\frac{v_{i}}{v_{0}}=\;\frac{1}{\left(1+\;\frac{\left[I\right]}{{IC}_{50}}\right)}$$

where *v_i_* is the enzyme activity in the presence of inhibitor, *v_0_* the activity in the absence of inhibitor, pI=−log [Inhibitor] in M and pIC_50_ = −log IC_50_ in M. All experiments were performed in at least triplicate.

For substrate competitive inhibitors, Equations (3) shows the relation between IC_50_ and *K_i_* values based on the fixed concentration of substrate used and the enzymes *K_m_* value for the substrate:

(3) $${\mathrm{IC}}_{50}=K_{i}\;(1+[S]/K_{m})$$

### Quenching experiments

Some of the catechol and biphosphate derivatives showed a concentration dependent fluorescence at wavelengths used for the McaPLGL(Dpa)AR-NH_2_ and McaRPPGFSAFK(Dnp)-OH substrates. To determine to which extent these derivatives could quench the time dependent enzymatic increase in the fluorescence product of the processed substrate, quenching experiments were performed as described previously^58^. Briefly, the fluorescence (*λ*_ex_=320 nm, *λ*_em_=405 nm, slit width = 10 nm) of various concentrations of the fluorescent product of the substrate McaPLGL(Dpa)AR-NH_2_, McaPL-OH (0–100 nM), was determined in absence and presence of various concentrations of the catechol and bisphosphonate derivatives (0–100 µM). Primary and secondary plots were used to determine whether the catechol and bisphosphonate derivatives quenched the McaPL-OH fluorescence.

### Docking

The internal Coordinate Mechanics (ICM) program version^68^ was used for docking of catechol containing compounds (ML32, BF471, ML33, BF489, BF486, BF482, BF466, MT336) and bisphosphonate containing compounds (RC14, LS4, RC2, MT242, GD16, ML45, MT363) into the target proteases. The X-ray crystal structures of PLN (PDB-code:1u4g), TLN (PDB-code: 5dpe), MMP-9 (PDB-code: 5cuh, ALN (PDB-code: 1bqb) and MMP-14 (PDB-code:1bqq) were collected from the PDB database and used for docking. Crystallographic water molecules were removed along with the co-crystallized small molecule inhibitors. Hydrogen atoms were added and optimised using the ECEPP/3 force field before the structures were refined and minimised. The various inhibitors were built using ICM and minimised before docking. The binding modes of the inhibitors in the X-ray structure complexes PLN (1u4g), TLN (5dpe) and MMP-9 (5cuh) were used to define the binding pocket for docking into these enzymes, using grid maps that included all amino acids within 5 Å of the cocrystallized inhibitors. However, X-ray crystal structures with small molecule inbitors were not available for ALN and MMP-14. For ALN, the X-ray structure without inhibitor (1bqb) was superimposed with the PLN complex (1u4g) and the inhibitor in the PLN complex was used to create docking grids including all amino acids within 5 Å of the PLN inhibitor. For MMP-14, the X-ray crystal structure of MMP-8 with the inhibitor BF471 (PDB id: 5h8x) was superimposed with the MMP-14 structure in complex with TIMP-2 (1bqq) and binding mode of BF471 in MMP-8 was used to create docking grids within 5 Å of BF471. After ceating grid maps, semi-flexible docking was performed where the enzymes were kept rigid while the ligands were structurally flexible. Each docking was run in three parallels. Ligand conformer sampling *in vacuo* and Monte Carlo global energy optimisation were used to generate docking poses^69^, while the poses were scored using the Virtual Ligand Scoring (VLS) module of the ICM program. The VLS scoring function uses steric, entropic, hydrogen bonding, hydrophobic and electrostatic terms to calculate the score and also includes a correction term proportional to the number of atoms in the ligand to avoid bias towards larger ligands^70^.
